# Association between Dry Eye Disease, Air Pollution and Weather Changes in Taiwan

**DOI:** 10.3390/ijerph15102269

**Published:** 2018-10-16

**Authors:** Jia-Yu Zhong, Yuan-Chieh Lee, Chia-Jung Hsieh, Chun-Chieh Tseng, Lih-Ming Yiin

**Affiliations:** 1 Department of Public Health, Tzu Chi University, 701, Sec. 3, Zhongyang Road, Hualien City 97004, Taiwan; 105324101@gms.tcu.edu.tw (J.-Y.Z.); cjhsieh@mail.tcu.edu.tw (C.-J.H.); tsengcc@mail.tcu.edu.tw (C.-C.T.); 2 Department of Ophthalmology and Visual Science, Tzu Chi University, 701, Sec. 3, Zhongyang Road, Hualien City 97004, Taiwan; yuanchieh.lee@gmail.com; 3 Department of Ophthalmology, Buddhist Tzu Chi General Hospital, 707, Sec. 3, Zhongyang Rd., Hualien City 97002, Taiwan

**Keywords:** air pollution, dry eye disease, relative humidity, Taiwan, temperature, traffic emission

## Abstract

Dry eye disease (DED) has become a common eye disease in recent years and appears to be influenced by environmental factors. This study aimed to examine the association between the first occurrence of DED, air pollution and weather changes in Taiwan. We used the systematic sampling cohort database containing 1,000,000 insureds of the National Health Insurance of Taiwan from 2004 to 2013, and identified a total of 25,818 eligible DED subjects. Environmental data, including those of air pollutants, temperature and relative humidity, were retrieved from the environmental monitoring stations adjacent to subjects’ locations of clinics as exposure information. We applied the case-crossover design, which used the same subjects experiencing exposures on diagnosis days as cases and those on other days as controls. The descriptive statistics showed that the first occurrences of DED were the most for the elderly by age (53.6%), women by gender (68.9%), and spring by season (25.9%). Multivariate conditional logistic regression analyses indicated that carbon monoxide (CO), nitrogen dioxide (NO_2_), and temperature were positively associated with DED (*p* < 0.05), while relative humidity was negatively related (*p* < 0.001). Because CO and NO_2_ together are considered a surrogate of traffic emission, which is easier to control than the uprising temperature, it is suggested that efficient management and control of traffic emission may lower the probability of DED occurrence.

## 1. Introduction

Dry eye disease (DED) has been considerable around the world in the recent decades. DED was initially considered “a disorder of the tear film due to tear deficiency or excessive tear evaporation” in 1995, and was later defined as “a multifactorial disease of the tears and ocular surface” by the first Tear Film and Ocular Surface Society Dry Eye Workshop (TFOS DEW I) in 2007; after gaining years of clinical evidence TFOS DEW II revised the definition to “a multifactorial disease of the ocular surface characterized by a loss of homeostasis of the tear film, and accompanied by ocular symptoms” in 2017 [1]. Because the definition of DED was under development, the diagnostic procedure around the world might not be standardized; thus, the DED prevalence reported by a number of studies conducted in various countries was quite different, ranging from 4.3% to 34% [2,3,4,5,6,7,8,9]. Despite the diverse diagnostic procedure of DED, a number of studies have indicated several risk factors in common, including age and gender [4,10,11,12,13]. It is summarized that the prevalence increases with age, women suffer the disease more than men, and the occurrence is seemingly more common in Asians than in other races in the TFOS DEW II Epidemiology Report [13]. In addition, contact lens wear has been found to be associated with DED [13,14,15,16].

As physical contact (e.g., wearing contact lens) with the eyes could have an impact on DED development, contact of air pollutants may have similar effects. Since air pollution has been confirmed to be associated with allergic conjunctivitis [17,18] and respiratory diseases, including asthma and allergic rhinitis [19,20,21,22], it is of interest to determine whether air pollution is also related to DED. In the literature review, thus far only two Korean studies showed a significant relationship between air pollution and DED, albeit with inconsistent air pollutants [23,24]. In this study, we would like to examine whether air pollution and weather change were associated with DED in Taiwan, and which environmental factors were significantly linked to the disease.

We took advantage of data from the National Health Insurance of Taiwan, which provided DED identified patients with useful information for linkage with the environmental monitoring data. The environmental data were measurements of air pollutants, such as carbon monoxide (CO), nitrogen dioxide (NO_2_), ozone (O_3_), particulate matter with aerodynamic diameter ≤2.5 and 10 µm (PM_2.5_, PM_10_), and sulfur dioxide (SO_2_), and meteorological recordings, including relative humidity (RH) and temperature. To the best of our knowledge, this is the first study to examine the association between air pollution, weather changes and DED in Taiwan; the result should be valuable to the general public for understanding the link between eye health and environmental degradation. 

## 2. Materials and Methods 

### 2.1. Inclusion of Subjects

The systematic sampling cohort database of 1,000,000 insureds of the National Health Insurance of Taiwan from 2004 to 2013 provided eligible subjects for this study. Diagnoses were recorded according to the International Classification of Diseases, the 9th Revision, Clinical Modification (ICD-9-CM). Individuals who were diagnosed with ICD-9-CM codes of 370.33 and/or 375.15 and given prescriptions with S01XA18 and/or A01XA20, the codes of the Anatomical Therapeutic Chemical Classification System, were identified as DED patients. Those who were diagnosed with the ICD-9-CM code of 710.2 (i.e., Sjögren’s syndrome) were excluded, because of a different origin of dry eye symptoms. There were 52,552 people diagnosed as DED patients during the study period; after removal of patients with Sjögren’s syndrome (5645), the patient number dropped to 46,907. Also, those without available environmental monitoring data or sufficient information were also removed from the database, and the final number of study subjects was 25,818 patients. The first DED diagnosis of each subject was used to examine the association with environmental factors in the study, which was granted for exempt review by the Research Ethics Committee of Tzu Chi General Hospital (No: IRB107-70-B, approved on 11 April 2018) for use of the secondary data with personal identification being removed. 

### 2.2. Environmental Monitoring Data

Environmental monitoring stations adjacent to or sharing the same district codes with the subjects’ clinics or hospitals were the sources of the environmental data. Of the available 76 environmental monitoring stations, 73 were matched with the DED subjects. Concentrations of air pollutants, CO, NO_2_, O_3_, PM_2.5_, PM_10_, and SO_2_, and meteorological data, RH and temperature, during the 10-year span were retrieved from the website of Taiwan Air Quality Network (http://taqm.epa.gov.tw/taqm/en/default.aspx). The daily average of each pollutant or meteorological factor was used in the analysis except ozone using the 8-h average, because of O_3_ formation occurring during the diurnal period of a day. 

### 2.3. Data Management and Analysis

Data were firstly categorized into groups by age, gender and season to examine whether these factors might differentiate the distribution of DED. To ascertain the representativeness of the selected subjects (25,818), we compared the patient profile to that of all DED subjects (46,907) using statistical tests (e.g., *t*-test, chi-square test). Environmental data were graphed to find any significant changes over time. We performed a Spearman correlation analysis for air pollutants and meteorological factors to understand the relations among these environmental factors. 

To find out the association between the first occurrence of DED, air pollution and weather changes, we applied the case-crossover design, which was useful when the risk factor/exposure was transient [25]. This design intends to use the same subjects for cases and controls, with the former at the onset of disease and the latter at other times. In this study, exposure for each case was the environmental data measured on the day of the first DED diagnosis, and exposure for control was the average of environmental data on four different days, which were one and two weeks before and after the onset of DED. 

Univariate conditional logistic regression analyses were conducted to learn the significance of the association between each air pollutant or meteorological factor and DED, whereas multivariate analyses were used to identify all significant factors that were associated with DED. Only one of the highly correlated air pollutants and meteorological factors (ρ > 0.8) was selected to enter a single multivariate analysis due to collinearity. Data of the environmental factors were directly used for conditional logistic regression analyses, except those with relatively large variations were converted to levels containing 10 units (e.g., ppb, µg/m^3^). Because of the case-crossover design where cases and controls were the same subjects with identical ages and genders and with days of exposure close to one another, adjustment for covariates (i.e., age, gender, season) was not necessary for the analysis. All the statistical analyses were performed using SAS 9.4 (SAS Institute Inc., Cary, NC, USA). 

## 3. Results

The descriptive statistics for study subjects and all DED patients is listed in Table 1. The average ages for both groups were around 51 years with no significant difference (*p* = 0.300), and the data categorized by gender and season also indicated similar patterns with no significant differences between study subjects and all DED patients, indicating excellent representativeness shown by study subjects. It appeared that people younger than 18 years were rarely diagnosed as DED patients (1.8%), but the percentage of DED occurrences went up with increasing age. Other than age, a gender difference was observed with females possessing almost 70% of the diagnoses. Seasonal variation of DED occurrence did not seem to be as obvious as the age or gender difference; however, the percentage in winter was the lowest and the difference in that from any other season was statistically significant (*p* < 0.001, not shown in table). It is suggested that the occurrence of DED may have been age-, gender- and season-specific. 

**Figure 1:**
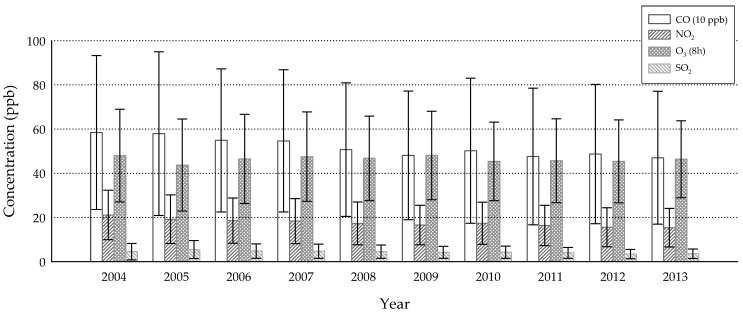
Annual mean concentrations of gaseous air pollutants (CO, NO_2_, O_3_, SO_2_) from 2004 to 2013 (error bars denote standard deviations), derived from 73 of 76 monitoring sites.

**Figure 2:**
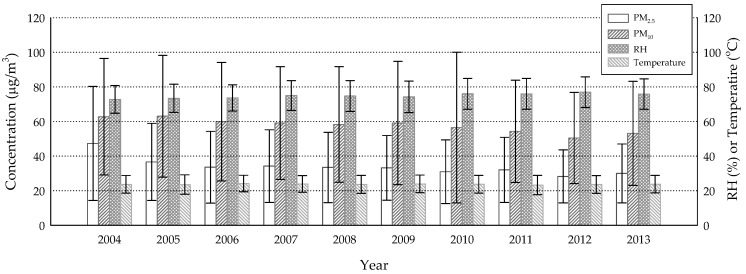
Annual averages of particulate matter with aerodynamic diameter ≤2.5 and 10 µm (PM_2.5_ and PM_10_) concentrations, relative humidity (RH) and temperature from 2004 to 2013 (error bars denote standard deviations), derived from 73 of 76 monitoring sites.

The annual averages of air pollution concentrations and meteorological parameters were shown in Figure 1 and Figure 2. The annual mean concentrations of CO, NO_2_ and SO_2_ were likely under Taiwan’s standards for ambient air quality, most of the time (9 ppm for the 8-h average, 50 ppb for the annual average, 100 ppb for the daily average, respectively); the others, however, were of concern. Although PM_2.5_ appeared to decrease over the years, every annual mean concentration exceeded the standard (15 µg/m^3^), suggesting an urgent need for this portion of pollution control. As for O_3_ and PM_10_, the concentrations were not far below the standards (60 ppb for the 8-h average, 65 µg/m^3^ for the annual average, respectively), indicating occasional occurrences of over-the-standard cases. Temperature and RH represented the typical sub-tropical climate over an island with means slightly above 20 °C and 70%, respectively. The highly correlated air pollutants were CO/NO_2_ (ρ = 0.828) and PM_2.5_/PM_10_ (ρ = 0.870) (Table 2), suggesting that they might have shared the same emission sources. It was interesting that RH was negatively correlated with all air pollutants. A reasonable explanation may be that high humidity usually comes with a shower that washes out certain levels of ambient air pollutants.

Based on the variations of air pollutants and meteorological factors, we decided to convert 10 units to one level for NO_2_, O_3_, PM_2.5_, PM_10_, and RH for the conditional logistic regression analyses. Each environmental factor was significantly related to the first occurrence of DED in the univariate analysis (*p* < 0.001, not shown in table), with an odds ratio (OR) larger than 1.0 except that for RH. It is suggested that low humidity may have favored the onset of DED. Multivariate analyses, conducted separately due to collinearity, indicated that the significant factors were CO or NO_2_, RH and temperature (Table 3). Every 1-ppm increase of CO accounted for an additional 10.5–11.6% of DED occurrence, whereas every 10-ppb increment of NO_2_ was related to additional 6.8–7.5% of DED occurrence. Temperature was associated with a relatively small margin of increase in DED occurrence (~1%) per degree Celsius increment. In contrast, every 10% increment of RH was related to approximately 6.7% reduction in DED occurrence, suggesting that moisture in the air might have mitigated the DED symptoms.

## 4. Discussion

As summarized in the TFOS DEWS II Epidemiology Report [13], the risk factors of age and gender were observed in this study, as well as in a number of previous studies [4,10,11,12,13]. Aging as a risk factor of DED is probably due to “normal changes in tear production and characteristics associated with advancing age”, as suggested by Moss et al. [12]. Another study indicated that an increase of tear evaporation by any disorder (e.g., Meibomian gland dysfunction) resulted in increased tear film osmolarity, which led to the symptoms of DED [26]. Compared with the data shown by an American study [4], our result had a relatively high percentage in the age group of 18–49 years (44% vs. 28%), indicating that DED commonly occurred to the younger generation in Taiwan. We propose that use of contact lenses might contribute to this early onset of DED [13,14,15,16], and overuse of mobile phones and tablets in the modern life style might be another factor. Further studies are required to validate this issue.

As for the higher prevalence in women, it is likely that sex hormones are effective in inducing DED. It was said in a review paper that androgen enhanced Meibomian gland function, which secreted oils onto the ocular surface keeping the tears from evaporation, and that estrogen and progesterone might antagonize the effect of androgen on Meibomian gland function [27]. Although the effects of estrogen and progesterone on the ocular surface are not well understood, it is suggested that estrogen may result in inflammation of the ocular surface, which underlies DED. Women, especially the elderly, are therefore at a high risk of DED, as shown in this study.

The highly correlated pollutants (i.e., CO/NO_2_, and PM_2.5_/PM_10_) may have shared the same sources. Traffic emission was likely the major source of CO and NO_2_ because they were simultaneously produced by incomplete combustion inside motor engines. By definition, PM_2.5_ was part of PM_10_, and certainly the high correlation between both was expected. However, both PM_2.5_ and PM_10_ could have originated from a variety of sources, including combustion, the photochemical smog reaction and sandstorms. In the multivariate conditional logistic regression analyses, we found that CO or NO_2_ was associated with DED, but PM_2.5_ or PM_10_ was not. It is likely that traffic emission, specifically surrogated by CO and NO_2_, is related to DED, whereas nonspecific pollutions represented by PM_2.5_ or PM_10_ fail to be relevant due to the complexity in sources.

The association between CO or NO_2_ and DED suggested that vehicle exhaust might have had an impact on the onset of DED. A commentary paper indicated that diesel exhaust, one of the much-concerned traffic emissions, containing potent oxidants resulted in inflammation on the ocular surface, and that NO_2_ itself could acidify the tears and led to reduced tear break-up time [28]. Both symptoms were commonly observed in the diagnosis of DED. Later, a study indicated greater tear film instability and more discomfort symptoms reported by subjects exposed to high levels of NO_2_ than those exposed to low levels of NO_2_, confirming the effect of traffic-derived air pollution on the ocular surface [29]. In addition to our result, a Korean study also showed a significant role of NO_2_ in relation to DED [23]. On the other hand, a previous study showed a significant association between DED and allergic rhinitis with positive skin-prick test results, indicating that DED could also be an allergic disease [30]. Allergic rhinitis has been found to be associated with CO and NO_2_ in several studies [19,21,31]; with the significantly associated air pollutants in common, it is suggested that DED, similar to allergic rhinitis, may be at least in part affected by allergens and air pollutants. Table 1 shows the highest percentage occurring in spring (~26%), which is in support of this suggestion.

Meteorological factors, RH and temperature, were both found related to DED but in opposite directions. A negative association between RH and DED, in agreement with both Korean studies [23,24], suggested that the moisture in the air might have helped sustain tear film on the ocular surface. An animal study using a mouse model indicated that exposure to a low-humidity environment for days resulted in alterations in tear secretion and goblet cell density, and acquisition of dry eye symptoms [32]. A human study testing effects of one-hour exposure to a dry environment on tear film indicated adverse effects on evaporation rate, lipid layer thickness, stability, and production of tears, compared to that of exposure to a normal environment [33]. As for temperature, our result suggested that elevated temperatures might have favored the occurrence of DED. A possible reasonable explanation is that high temperatures resulted in increased tear evaporation and led to dry eye symptoms. A human study was in support of this assumption showing a high tear evaporation rate at a high room temperature [34]. Interestingly, that study also showed increased lipid layer thickness and break-up time of tears at the temperature, compared to those at relatively low ones; the increase in lipid layer thickness or break-up time, however, was considered a protection factor from DED. The paradox is not too confusing, because the low temperatures tested in that study were below 15 °C, which were rarely seen in the year-round sub-tropical Taiwan; that is, only the result of increased tear evaporation rate was applied to people in Taiwan most of the time. Additionally, the paradox may explain why temperature rise is associated with limited chances of increase in DED occurrence (~1%/°C). Based on these results, maintaining appropriate humidity and temperature in the ambient air is recommended for eye health, especially for that of DED patients. 

It appears that air pollution from traffic emissions is associated with DED and other allergic diseases; thus, lowering the pollution levels of vehicle exhaust should be a necessary approach to mitigate these health problems. A report says that a number of countries around the world are working on banning the production and sales of gasoline and diesel cars, and meanwhile on promoting of electric vehicles to make their air clean [35]. By doing so, an improvement in the prevalence of DED and other allergic diseases will be expected in the near future. Temperature rise as a result of the ongoing climate change is also of concern to DED patients because of high tear evaporation rates. As well, climate change may prolong the allergic season (i.e., spring and early summer) [36], and result in more occurrences of allergen-initiated DED than in the past. Since this global phenomenon is not easy to reverse, care has to be taken for those who suffer dry eye symptoms.

This study contains several limitations. Firstly, not all identified DED patients were used as study subjects because of the availability of environmental monitoring stations. Fortunately, the characteristics of study subjects resembled that of all patients without significant differences between them (Table 1), suggesting that the study subjects should be sufficient to represent all patients. Secondly, use of databases with removal of identification for epidemiological studies is unable to access personal information that may be influential. To avoid such difficulties in the analysis, we applied the case-crossover design, using subjects themselves for cases and controls; thus, effects caused by personal practices or habits could be omitted. Thirdly, these results are derived from Taiwanese environmental and health insurance data, and whether they could be extended for other countries remains uncertain. Therefore, the information given herein should be used with caution for any further purposes. 

## 5. Conclusions

DED appeared to happen the most to the elderly older than 50 years by age, women by gender, and spring by season, in Taiwan during the period from 2004 to 2013. Traffic-related pollutants (CO, NO_2_) and temperature were found to be positively associated with DED, while RH was negatively related. As the uprising temperature resulting from climate change is difficult to manage, control of traffic emission is relatively easy in comparison. Therefore, we should make great effort to phase out fossil fuel powered vehicles, and make the air we breathe cleaner and our health better.

## Figures and Tables

**Table 1:** Data for study subjects and all patients at the first occurrence of dry eye disease (DED) by age, gender and season during 2004–2013.

	Study Subjects	All Patients	*p*-Value
Total	25,818	46,907	
Age (Mean ± SD)	51.1 ± 17.7	51.3 ± 17.5	0.300 ^a^
	n (%)	n (%)	
Age			0.285 ^b^
<18	472 (1.8)	867 (1.9)	
18~49	11,520 (44.6)	20,644 (44.0)	
≥50	13,826 (53.6)	25,396 (54.1)	
Gender			0.687 ^b^
Male	8021 (31.1)	14,505 (30.9)	
Female	17,797 (68.9)	32,402 (69.1)	
Season			0.521 ^b^
Spring	6684 (25.9)	12,282 (26.2)	
Summer	6561 (25.4)	11,931 (25.4)	
Fall	6407 (24.8)	11,711 (25.0)	
Winter	6166 (23.9)	10,983 (23.4)	

^a^ two sample *t*-test; ^b^ chi-square test.

**Table 2:** Spearman correlation among daily air pollutants and meteorological factors.

	CO	NO_2_	O_3_ (8h)	PM_2.5_	PM_10_	SO_2_	RH	Temperature
CO	1							
NO_2_	0.828	1						
O_3_ (8h)	0.025	0.074	1					
PM_2.5_	0.429	0.533	0.503	1				
PM_10_	0.372	0.473	0.461	0.870	1			
SO_2_	0.346	0.529	0.160	0.480	0.455	1		
RH	–0.123	–0.198	–0.297	–0.281	–0.328	–0.232	1	
Temperature	–0.186	–0.216	0.115	–0.164	–0.163	0.019	–0.108	1

All correlations are significant, *p* < 0.001.

**Table 3:** Multivariate conditional logistic regression analyses between DED, air pollutants and meteorological factors.

	Model 1 ^a^		Model 2 ^b^		Model 3 ^c^		Model 4 ^d^	
	OR (95%CI)	*p*-Value	OR (95%CI)	*p*-Value	OR (95%CI)	*p*-Value	OR (95%CI)	*p*-Value
CO (ppm)	1.116 (1.026, 1.214)	0.010	-	-	1.105 (1.004, 1.216)	0.042	-	-
NO_2_ (10 ppb)	-	-	1.068 (1.037, 1.100)	<0.001	-	-	1.075 (1.040, 1.111)	<0.001
O_3_ 8h (10 ppb)	1.000 (0.990, 1.011)	0.932	0.998 (0.998, 1.009)	0.725	1.000 (0.989, 1.011)	0.960	0.997 (0.986, 1.008)	0.616
PM_10_ (10 μg/m^3^)	1.001 (0.994, 1.009)	0.717	1.000 (0.992, 1.007)	0.920	-	-	-	-
PM_2.5_ (10 μg/m^3^)	-	-	-	-	1.006 (0.991, 1.022)	0.422	1.001 (0.986, 1.016)	0.900
SO_2_ (ppb)	1.004 (0.996, 1.012)	0.284	0.998 (0.990, 1.006)	0.621	1.006 (0.998, 1.015)	0.148	1.000 (0.991, 1.008)	0.923
RH (10%)	0.935 (0.916, 0.954)	<0.001	0.930 (0.910, 0.949)	<0.001	0.936 (0.916, 0.956)	<0.001	0.929 (0.909, 0.949)	<0.001
Temperature (°C)	1.008 (1.002, 1.013)	0.004	1.010 (1.005, 1.015)	<0.001	1.008 (1.003, 1.014)	0.003	1.011 (1.005, 1.016)	<0.001

^a^ NO_2_ and PM_2.5_ excluded from the model due to collinearity with CO and PM_10_, respectively; ^b^ CO and PM_2.5_ excluded from the model due to collinearity with NO_2_ and PM_10_, respectively; ^c^ NO_2_ and PM_10_ excluded from the model due to collinearity with CO and PM_2.5_, respectively; ^d^ CO and PM_10_ excluded from the model due to collinearity with NO_2_ and PM_2.5_, respectively. Odds ratio (OR).

## References

[1] Craig J.P., Nichols K.K., Akpek E.K., Caffery B., Dua H.S., Joo C.K., Liu Z., Nelson J.D., Nichols J.J., Tsubota K. (2017). TFOS DEWS II definition and classification report. Ocul. Surf..

[2] Lee A.J., Lee J., Saw S.M., Gazzard G., Koh D., Widjaja D., Tan D.T. (2002). Prevalence and risk factors associated with dry eye symptoms: A population based study in Indonesia. Br. J. Ophthalmol..

[3] Uchino M., Nishiwaki Y., Michikawa T., Shirakawa K., Kuwahara E., Yamada M., Dogru M., Schaumberg D.A., Kawakita T., Takebayashi T. (2011). Prevalence and risk factors of dry eye disease in Japan: Koumi study. Ophthalmology.

[4] Farrand K.F., Fridman M., Stillman I.O., Schaumberg D.A. (2017). Prevalence of diagnosed dry eye disease in the United States among adults aged 18 years and older. Am. J. Ophthalmol..

[5] Jie Y., Xu L., Wu Y.Y., Jonas J.B. (2009). Prevalence of dry eye among adult Chinese in the Beijing Eye Study. Eye.

[6] Lin P.-Y., Tsai S.-Y., Cheng C.-Y., Liu J.-H., Chou P., Hsu W.-M. (2003). Prevalence of dry eye among an elderly Chinese population in Taiwan. Ophthalmology.

[7] Schaumberg D.A., Dana R., Buring J.E., Sullivan D.A. (2009). Prevalence of dry eye disease among US men: Estimates from the Physicians’ Health Studies. Arch. Ophthalmol..

[8] Lekhanont K., Rojanaporn D., Chuck R.S., Vongthongsri A. (2006). Prevalence of dry eye in Bangkok, Thailand. Cornea.

[9] Schaumberg D.A., Sullivan D.A., Buring J.E., Dana M.R. (2003). Prevalence of dry eye syndrome among US women. Am. J. Ophthalmol..

[10] Paulsen A.J., Cruickshanks K.J., Fischer M.E., Huang G.H., Klein B.E., Klein R., Dalton D.S. (2014). Dry eye in the beaver dam offspring study: Prevalence, risk factors, and health-related quality of life. Am. J. Ophthalmol..

[11] Ahn J.M., Lee S.H., Rim T.H., Park R.J., Yang H.S., Kim T.I., Yoon K.C., Seo K.Y. (2014). Prevalence of and risk factors associated with dry eye: The Korea National Health and Nutrition Examination Survey 2010–2011. Am. J. Ophthalmol..

[12] Moss S.E., Klein R., Klein B.E. (2000). Prevalence of and risk factors for dry eye syndrome. Arch. Ophthalmol..

[13] Stapleton F., Alves M., Bunya V.Y., Jalbert I., Lekhanont K., Malet F., Na K.S., Schaumberg D., Uchino M., Vehof J. (2017). TFOS DEWS II Epidemiology Report. Ocul. Surf..

[14] Man R.E.K., Veerappan A.R., Tan S.P., Fenwick E.K., Sabanayagam C., Chua J., Leong Y.Y., Wong T.Y., Lamoureux E.L., Cheng C.Y. (2017). Incidence and risk factors of symptomatic dry eye disease in Asian Malays from the Singapore Malay Eye Study. Ocul. Surf..

[15] Bakkar M.M., Shihadeh W.A., Haddad M.F., Khader Y.S. (2016). Epidemiology of symptoms of dry eye disease (DED) in Jordan: A cross-sectional non-clinical population-based study. Cont. Lens Anterior Eye.

[16] Lemp M.A., Bielory L. (2008). Contact lenses and associated anterior segment disorders: Dry eye disease, blepharitis, and allergy. Immunol. Allergy Clin. N. Am..

[17] Mimura T., Ichinose T., Yamagami S., Fujishima H., Kamei Y., Goto M., Takada S., Matsubara M. (2014). Airborne particulate matter (PM_2.5_) and the prevalence of allergic conjunctivitis in Japan. Sci. Total Environ..

[18] Hong J., Zhong T., Li H., Xu J., Ye X., Mu Z., Lu Y., Mashaghi A., Zhou Y., Tan M. (2016). Ambient air pollution, weather changes, and outpatient visits for allergic conjunctivitis: A retrospective registry study. Sci. Rep..

[19] Chung H.Y., Hsieh C.J., Tseng C.C., Yiin L.M. (2016). Association between the first occurrence of allergic rhinitis in preschool children and air pollution in Taiwan. Int. J. Environ. Res. Public Health.

[20] Guo Y.L., Lin Y.C., Sung F.C., Huang S.L., Ko Y.C., Lai J.S., Su H.J., Shaw C.K., Lin R.S., Dockery D.W. (1999). Climate, traffic-related air pollutants, and asthma prevalence in middle-school children in Taiwan. Environ. Health Perspect..

[21] Lee Y., Shaw C., Su H., Lai J., Ko Y., Huang S.-L., Sung F.-C., Guo Y. (2003). Climate, traffic-related air pollutants and allergic rhinitis prevalence in middle-school children in Taiwan. Eur. Respir. J..

[22] Brauer M., Hoek G., Van Vliet P., Meliefste K., Fischer P.H., Wijga A., Koopman L.P., Neijens H.J., Gerritsen J., Kerkhof M. (2002). Air pollution from traffic and the development of respiratory infections and asthmatic and allergic symptoms in children. Am. J. Respir. Crit. Care Med..

[23] Hwang S.H., Choi Y.H., Paik H.J., Wee W.R., Kim M.K., Kim D.H. (2016). Potential importance of ozone in the association between outdoor air pollution and dry eye disease in South Korea. JAMA Ophthalmol..

[24] Um S.B., Kim N.H., Lee H.K., Song J.S., Kim H.C. (2014). Spatial epidemiology of dry eye disease: Findings from South Korea. Int. J. Health Geogr..

[25] Maclure M. (1991). The case-crossover design: A method for studying transient effects on the risk of acute events. Am. J. Epidemiol..

[26] Gilbard J.P. (1994). Human tear film electrolyte concentrations in health and dry-eye disease. Int. Ophthalmol. Clin..

[27] Truong S., Cole N., Stapleton F., Golebiowski B. (2014). Sex hormones and the dry eye. Clin. Exp. Optom..

[28] Leonardi A., Lanier B. (2008). Urban eye allergy syndrome: A new clinical entity?. Curr. Med. Res. Opin..

[29] Novaes P., Saldiva P.H., Matsuda M., Macchione M., Rangel M.P., Kara-Jose N., Berra A. (2010). The effects of chronic exposure to traffic derived air pollution on the ocular surface. Environ. Res..

[30] Yenigun A., Dadaci Z., Sahin G.O., Elbay A. (2016). Prevalence of allergic rhinitis symptoms and positive skin-prick test results in patients with dry eye. Am. J. Rhinol. Allergy.

[31] Hwang B.-F., Jaakkola J.J., Lee Y.-L., Lin Y.-C., Guo Y.-l.L. (2006). Relation between air pollution and allergic rhinitis in Taiwanese schoolchildren. Respir. Res..

[32] Barabino S., Shen L., Chen L., Rashid S., Rolando M., Dana M.R. (2005). The controlled-environment chamber: A new mouse model of dry eye. Investig. Ophthalmol. Vis. Sci..

[33] Abusharha A.A., Pearce E.I. (2013). The effect of low humidity on the human tear film. Cornea.

[34] Abusharha A.A., Pearce E.I., Fagehi R. (2016). Effect of ambient temperature on the human tear film. Eye Contact Lens.

[35] Cable News Network (CNN) (2017). These Countries Want to Ban Gas and Diesel Cars. Https://money.cnn.com/2017/09/11/autos/countries-banning-diesel-gas-cars/index.html.

[36] Beggs P.J., Bambrick H.J. (2005). Is the global rise of asthma an early impact of anthropogenic climate change?. Environ. Health Perspect..

